# Positive mental health of patients at a psychiatric hospital, Gauteng province, South Africa

**DOI:** 10.4102/sajpsychiatry.v29i0.2016

**Published:** 2023-06-23

**Authors:** Satya Sai Ram Kumar Gulla, Elvera Helberg, Rajesh Vikram Vagiri

**Affiliations:** 1 Department of Public Health Pharmacy and Management, School of Pharmacy, Sefako Makgatho Health Sciences University, Pretoria, South Africa; 2 Department of Pharmacy, Faculty of Health Sciences, University of Limpopo, Sovenga, South Africa

**Keywords:** mental health, psychological well-being, positive mental health, multi-dimensional, mental health care users

## Abstract

**Background:**

Positive mental health (PMH) forms the basis of well-being and positive mind set, which includes a range of cognitive-emotional attributes and coping skills of an individual towards the family and society. Assessment of PMH in psychiatric patients is critical in understanding their needs, improving mental health and the treatment of their illnesses.

**Aim:**

To investigate the levels of PMH among patients attending the outpatient department at a public sector tertiary referral psychiatric hospital using the multidimensional PMH instrument.

**Setting:**

Adult psychiatric patients attending the outpatient department at a public sector tertiary referral hospital, Gauteng province, South Africa.

**Methods:**

A quantitative, cross-sectional and descriptive study was conducted using a multi-dimensional PMH instrument with a convenient sample of 346 outpatients who provided consent.

**Results:**

Females reported significantly high PMH scores (3.86 vs 3.6; *p* = 0.018) compared to males. Patients with higher education (Gr. 0–7 vs Gr. 8–12 vs Tertiary education, PMH scores 3.34 vs 3.75 vs 4.18; *p* < 0.001), being married (single vs married, 3.67 vs 3.81, *p* = 0.342) and employed (unemployed vs employed, 3.62 vs 3.97, *p* = 0.005) reported significantly high total PMH score and across various domains.

**Conclusion:**

The results of the study highlighted the multi-dimensionality of mental health and justified importance of evaluating the domains of PMH in mental health care users. Identifying the reasons for the deficits in the PMH domains and implementing coping strategies will improve the emotional and psychological well-being of patients.

**Contribution:**

Examining the PMH domains will assist healthcare workers intervene to improve the mental health of patients.

## Introduction

South Africa has been the lowest ranked country based on the mental well-being of its population, according to the Mental State of the World report, which further showcased the rapid decline of mental health in younger generations.^1^ South Africa’s National Mental Health Policy Framework and Strategic Plan 2013–2020 recommends reducing the burden of untreated mental health conditions through mental health prevention and promotion, as well as the integration of mental health services into general healthcare systems.^2^

The World Health Organization^3^ defines mental health as:

… a state of well-being in which every individual realizes his or her own potential, can cope with the normal stresses of life, can work productively and fruitfully, and is able to contribute to her or his community. (p. 19)

The concept of well-being relates to the integration of two approaches: the ‘hedonic’ and ‘eudaimonic’ approaches which, although distinct, are highly related subjective experiences. The hedonic approach includes positive effects such as happiness (pleasant and life satisfaction), and the eudaimonic framework includes personal functioning and social well-being (psychological and social functioning).^4^

The psychological well-being of individuals is a combination of various domains of positive self-esteem, mastery of the environment, good interpersonal relationships with others, continued growth and development, purposeful living and self-determination skills.^5^ The positive mental health (PMH) approach was first introduced by Jahoda^6^ as ‘individuals’ attitudes towards themselves in relation to autonomy, perception to reality and mastering their environment’. The PMH includes multiple dimensions of mental well-being, such as positive effect, contentment and psychological functioning, as well as the ability to accomplish them. Enhancing the psychological wellness and overall health of individuals experiencing mental illnesses is an essential component in mental health treatment.^7^

In evaluating the PMH, it is important to understand the mechanism by which significant elements and life occurrences impact the degree of positive consequences, and the advancement of adverse psychological aftermaths in patients is crucial. Investigating the PMH domains in patients with mental illnesses will help to identify their needs, help to treat medical ailments and improve their psychological health. Limited studies were conducted in advanced and developing countries on the influence of different domains of PMH on patients suffering with mental illnesses.^8^ By improving the psychological well-being, proficiency and resilience, of establishing supportive living conditions and settings, mental health promotes PMH.^9^

According to a review of the literature, no studies have been done in South Africa to evaluate the mental health and well-being of patients getting treatment for mental illnesses using a PMH instrument. There is a requirement to capacitate psychiatric patients for establishing and maintaining relationships, managing interpersonal relationships, offering and accepting emotional assistance, achieving personal development and self-determination and engaging in spiritual and religious traditions.^10^

The PMH provides an influential shielding aspect against mental disorders. Taking into consideration the fact that mental illnesses and PMH are integral parts of mental health, promotion and prevention are conceptually distinct; however, they apply similar approaches to mental health in general. While making good decisions for the promotion of public health, evidence-based prevention strategies inspired from systemic research should be applied. Identifying deficiencies and improving the patient’s PMH in South Africa are key components of this strategy.

This study investigated the level of PMH among patients attending the outpatient department at a tertiary referral psychiatric hospital in Gauteng province, South Africa, using the multidimensional PMH instrument developed and validated by Vaingankar et al.^11^

## Research methods and design

### Setting

The study was conducted at an outpatient department at a public sector tertiary referral psychiatric hospital situated in the West of Pretoria, Gauteng province, South Africa.

### Study design and population

The study used a descriptive cross-sectional study design and a quantitative methodology. Using Raosoft^®12^ to calculate sample size, 346 patients were chosen as a sample, with a 5% margin of error, a 95% confidence level and a response distribution and/or proportion of 50%. The following formula was used to determine the sample size (*n*):

$$n=N*(\text{X}+N-1),\text{where X}={\text{Z}}_{\alpha /2}{}^{2}-*p*(1-p)/{\text{MOE}}^{2}$$ [Eqn 1]

where:

Z_α/2_ = critical value of the normal distribution at α/2 (confidence level of 95%, α is 0.05 and the critical value is 1.96),

MOE = margin of error,

*p* = sample proportion and

*N* = population size.

### Study population

The study population included patients receiving treatment for psychiatric illnesses attending an outpatient department at a public sector tertiary referral psychiatric hospital. Approximately 2000 patients receive psychiatric services at the study site per month.

### Sample selection and recruitment

The convenience sampling procedure was utilised to recruit patients for the study. The participants of the study included adult patients (> 18 years) who are on treatment for psychiatric illness and who provided consent to take part in the study.

Exclusion criteria were applied to patients who did not provide consent to participate and who could not complete the questionnaire.

### Study instrument

The study used the PMH instrument containing multidimensions of various domains developed by Vaingankar et al.^11^ When comparing the levels of mental health among various populations, a PMH instrument is a self-administered tool that includes all significant and culturally appropriate domains of mental health. The PMH instrument has 57 measures including the pillars of mental health such as general coping, emotional support, spirituality, interpersonal skills, personal growth and autonomy, and global effect.

All the questionnaires and consent forms were made available in English, Afrikaans and Setswana as they are the predominant languages spoken by the outpatients at the study site. The English version was translated into Afrikaans and Setswana and then backtranslated into English. The translations were compared with the original version for validity. The questionnaire consisted of two sections.

Section 1: Sociodemographic information of respondents

Section 2: Positive mental health instrument consisting of 57 questions incorporating all six domains of mental health.

### Ethical considerations

This study’s ethical approval was acquired from Sefako Makgatho University Research Ethics Committee (SMUREC) (SMUREC/P/17/2021 PG). Permission from the Gauteng Department of Health and the Chief Executive Officer of the public sector tertiary referral psychiatric hospital to conduct research was obtained.

A patient information leaflet was provided to participants informing them of the objectives of the study. It was made clear that taking part in the study was optional and that participants had the right to leave at any time without having to offer a reason and that their identity would remain anonymous. Concerns by prospective participants regarding the study were clarified before the commencement of data collection. Participants provided written consent before their participation in the study. Adherence to ethical standards and ethical research practices was maintained. All records and data were kept in a safe and secured locker to maintain confidentiality.

### Data collection

Data were collected over a period of 12 weeks from March 2021 to June 2021. Participants who expressed their willingness to take part were recruited for this study. The questionnaires were self-administered after participants had given consent to take part in the study.

### Data entry and analysis

Responses from the questionnaires were exported to Microsoft Office Excel™ calculating the scores according to the scoring script provided by Vaingankar et al.^11^ and analysed using IBM’s Statistical Package for the Social Studies (SPSS) version 28.^13^ The statistical significance for the study was set at *p* ≤ 0.05 using two-sided tests. Differences in the mean scores for the total PMH and domains, as well as sociodemographic variables, were assessed using independent *t*-tests and analysis of variance. To control confounding effects, relationships between each PMH domain and sociodemographic variables were established using the multiple linear regression analysis.

## Results

The study’s 346 outpatient participants had an average age of 39.41 (standard deviation [s.d.]: 13.85) years. Table 1 displays the sociodemographic details of the study sample. Less than half (45.1%) of the patients were aged between 18 and 35 years (45.1%) and their level of education ranged between Grades 8 and 12 (49.7%). More than half (59.2%) of the patients were African males (58.9%) and living with the family (70.8%). Majority of the patients were unemployed (83.8%) and unmarried (93.0%).

**TABLE 1:** Study sample’s sociodemographic characteristics (*n* = 346).

Sociodemographic characteristics	Number of patients	Percentage
**Questionnaire language**		
English	242	69.9
Afrikaans	104	30.1
Setswana	0	0
**Gender**		
Male	205	59.2
Female	141	40.8
**Age (in years)**		
18–35	156	45.0
36–54	133	38.5
> 55	57	16.5
**Level of education**		
Gr. 0–7	117	33.8
Gr. 8–12	172	49.7
Tertiary education	57	16.5
**Living status**		
With family	245	70.8
Alone	62	17.9
With friends	39	11.3
**Employment**		
Unemployed	290	83.8
Employed	56	16.2
**Marital status**		
Single	322	93.0
Married	24	7.0
**Years on treatment**		
0–3	114	33.9
4–7	182	52.6
> 8	50	14.5

### Total positive mental health and domain-specific scores by sociodemographic variables

Table 2 displays the sociodemographic variable’s mean total PMH and domain-specific scores. Significant differences in the total PMH and domain scores were observed for several sociodemographic variables. Gender (*p* = 0.018), educational level (*p* < 0.001) and employment (*p* = 0.005) had a significant influence on the total PMH score. Patients who are female, employed and possessed tertiary education reported higher mean scores in most domains and total PMH scores. In case of the general coping domain, significant differences in mean scores were observed in the following sociodemographic variables: educational level (*p* < 0.001), living status (*p* = 0.0028) and employment (*p* = 0.036). Significant differences in mean scores with regard to the emotional support domain were observed with age (*p* = 0.007), educational level (*p* = 0.022) and living status (*p* < 0.001).

**TABLE 2:** Scores for each domain and the total positive mental health according to sociodemographic characteristics.

Sociodemographic characteristics	Total PMH		General coping		Emotional support		Spirituality		Interpersonal skills		Personal growth and autonomy		Global effect	
	Mean ± s.d.	*p*	Mean ± s.d.	*p*	Mean ± s.d.	*p*	Mean ± s.d.	*p*	Mean ± s.d.	*p*	Mean ± s.d.	*p*	Mean ± s.d.	*p*
**Language**		0.860		0.587		0.085		0.968		0.005*		0.095		0.552
English	3.68 ± 0.81		3.63 ± 0.89		3.92 ± 1.05		3.78 ± 1.41		3.75 ± 0.89		3.43 ± 1.11		3.61 ± 1.17	
Afrikaans	3.67 ± 0.68		3.69 ± 0.89		3.76 ± 0.86		3.78 ± 1.16		3.75 ± 0.74		3.15 ± 0.88		3.53 ± 1.16	
**Gender**		0.018*		0.321		0.459		0.644		1.037		0.531		0.167
Male	3.60 ± 0.74		3.61 ± 0.87		3.71 ± 0.93		3.52 ± 1.35		3.71 ± 0.79		3.43 ± 1.01		3.66 ± 1.18	
Female	3.85 ± 0.81		3.71 ± 0.92		4.17 ± 1.05		4.16 ± 1.24		3.81 ± 0.92		3.50 ± 1.10		3.49 ± 1.16	
**Age in years**		0.588		0.550		0.007*		0.067		0.483		0.946		0.969
18–35	3.70 ± 0.75		3.71 ± 0.84		4.02 ± 0.94		3.64 ± 1.29		3.80 ± 0.81		3.47 ± 1.06		3.58 ± 1.12	
36–54	3.63 ± 0.76		3.60 ± 0.86		3.68 ± 1.00		3.80 ± 1.40		3.68 ± 0.85		3.45 ± 1.02		3.59 ± 1.20	
> 55	3.75 ± 0.89		3.61 ± 1.10		4.06 ± 1.09		4.12 ± 1.29		3.78 ± 0.94		3.42 ± 1.08		3.62 ± 1.27	
**Education level**		< 0.001*		< 0.001*		0.022*		< 0.001*		< 0.001*		< 0.001*		< 0.001*
Gr. 0–7	3.34 ± 0.62		3.39 ± 0.75		3.71 ± 0.86		3.26 ± 1.10		3.38 ± 0.73		3.07 ± 0.85		3.32 ± 1.08	
Gr. 8–12	3.75 ± 0.78		3.73 ± 0.91		3.95 ± 1.05		3.87 ± 1.42		3.86 ± 0.81		3.51 ± 1.04		3.61 ± 1.15	
Tertiary	4.18 ± 0.75		3.94 ± 0.98		4.13 ± 1.05		4.59 ± 1.09		4.24 ± 0.86		4.08 ± 1.11		4.18 ± 0.75	
**Living status**		0.156		0.028*		< 0.001*		0.005*		0.754		0.314		0.213
With family	3.69 ± 0.80		3.59 ± 0.94		4.06 ± 0.98		3.85 ± 1.36		3.74 ± 0.86		3.43 ± 1.09		3.54 ± 1.17	
Alone	3.78 ± 0.77		3.92 ± 0.80		3.57 ± 1.13		3.93 ± 1.25		3.83 ± 0.91		3.63 ± 0.93		3.83 ± 1.24	
With friends	3.48 ± 0.60		3.58 ± 0.63		3.41 ± 0.60		3.12 ± 1.16		3.74 ± 0.62		3.36 ± 0.89		3.53 ± 1.02	
**Employment**		0.005*		0.036*		0.939		0.222		0.019*		< 0.001*		0.004*
Unemployed	3.62 ± 0.75		3.60 ± 0.89		3.90 ± 0.98		3.74 ± 1.33		3.70 ± 0.82		3.35 ± 1.02		3.50 ± 1.12	
Employed	3.97 ± 0.82		3.88 ± 0.88		3.91 ± 1.12		3.99 ± 1.41		4.02 ± 0.92		3.98 ± 1.01		4.06 ± 1.31	
**Marital status**		0.342		0.410		0.060		0.047*		0.953		0.541		0.892
Single	3.67 ± 0.76		3.64 ± 0.88		3.87 ± 0.98		3.73 ± 1.32		3.75 ± 0.83		3.44 ± 1.03		3.59 ± 1.15	
Married	3.81 ± 1.00		3.69 ± 1.02		4.28 ± 1.20		4.39 ± 1.50		3.74 ± 1.09		3.60 ± 1.22		3.62 ± 1.45	
**Years on treatment**		0.666		0.84		0.189		0.572		0.362		0.706		0.176
0–3	3.65 ± 0.66		3.67 ± 0.83		3.77 ± 0.88		3.67 ± 1.21		3.68 ± 0.70		3.52 ± 0.91		3.68 ± 1.13	
4–7	3.71 ± 0.81		3.66 ± 0.89		3.99 ± 1.02		3.83 ± 1.38		3.81 ± 0.88		3.44 ± 1.11		3.61 ± 1.19	
> 8	3.61 ± 0.88		3.58 ± 1.05		3.86 ± 1.17		3.85 ± 1.48		3.70 ± 1.01		3.38 ± 1.11		3.31 ± 1.18	

* *p* ≤ 0.05.

PMH, positive mental health; s.d., standard deviation; Gr., grade.

The educational level (*p* < 0.001), living status (*p* = 0.005) and marital status (*p* = 0.047) had a significant influence on the spirituality domain. In case of the interpersonal skills domain, a significant difference in mean scores was observed with the level of education (*p* < 0.001), employment (*p* = 0.019) and language of the questionnaire (*p* = 0.005). The personal growth and autonomy, and global effect domains showed significant differences (*p* = 0.05) in mean scores with levels of education and employment.

### Correlations between sociodemographic characteristics, total positive mental health and domain scores in the study population

The relationship between the total PMH and domains and the sociodemographic characteristics was determined using the general linear regression model.

The educational status was found to be significantly (*p* < 0.001) associated with the total PMH and all domains and was determined to be a predictor of PMH in the study population. Employment also had a significant influence on the total PMH (*p* = 0.005) and most domains, excluding the general coping and emotional support. The living status correlated with the emotional support, spirituality and global effect domains. Gender was found to be a predictor for the total PMH (*p* = 0.018), and marital status showed a significant (*p* = 0.047) association with the spirituality domain only. Table 3 illustrates the association between the sociodemographic characteristics and the total PMH and domains.

**TABLE 3:** Correlation between sociodemographic characteristics, total positive mental health and domains.

Sociodemographic characteristics	Total PMH		General coping		Emotional support		Spirituality		Interpersonal skills		Personal growth and autonomy		Global effect	
	ß	95% CI	ß	95% CI	ß	95% CI	ß	95% CI	ß	95% CI	ß	95% CI	ß	95% CI
**Language**														
English vs. Afrikaans	−0.01	−0.24, 0.21	0.08	−0.19, 0.35	−0.09	−0.39, 0.20	−0.08	−0.47, 0.31	−0.20*	−0.45, 0.54	0.20	−0.11, 0.51	−0.01	−0.36, 0.35
**Gender**														
Male vs. Female	0.23*	0.05, 0.40	0.20	−0.01, 0.41	0.42	0.01, 0.65	0.53	0.22, 0.83	0.13	−0.55, 0.33	0.15	−0.08, 0.39	−0.15	−0.43, 0.12
**Age**														
18–35 vs. 36–54 vs. > 55	−0.06	−0.18, 0.06	−0.12	−0.27, 0.02	−0.18*	−0.34, -0.02	0.05	−0.16, 0.26	−0.12	−0.26, 0.01	−0.47	−0.21, 0.12	0.13	−0.06, 0.33
**Education level**														
Gr. 0–7 vs. Gr. 8–12 vs. tertiary	0.40*	0.28, 0.51	0.28*	0.13, 0.42	0.22*	0.07, 0.38	0.62*	0.42, 0.83	0.41*	0.28, 0.54	0.45*	0.29, 0.61	0.34*	0.16, 0.53
**Living status**														
With family vs. alone vs. with friends	0.01	−0.138, 0.10	0.10*	−0.04, 0.24	0.26*	−0.42, -0.10	−0.11*	−0.32, 0.08	0.03	−0.09, 0.17	0.02	−0.14, 0.18	−0.01	−0.19, 0.19
**Employment**														
Unemployed vs. employed	0.18*	−0.03, 0.41	0.12*	−0.14, 0.39	0.01	−0.27, 0.31	0.09	−0.29, 0.47	0.14*	−0.09, 0.39	0.40*	0.09, 0.70	0.35*	0.002, 0.70
**Marital status**														
Single vs. married	0.08	−0.23, 0.39	0.05	−0.32, 0.43	0.22	−0.18, 0.64	0.29*	−0.25, 0.83	−0.06	−0.41, 0.27	0.07	−0.36, 0.50	−0.045	−0.54, 0.45
**Years of treatment**														
0–3 vs. 4–7 vs. > 8	0.01	−0.13, 0.10	−0.01	−0.16, 0.13	0.07	−0.08, 0.24	−0.01	−0.22, 0.20	0.03	−0.22, 0.16	−0.51	−0.22, 0.11	0.15	−0.34, 0.045

* *p* ≤ 0.05.

Note: Relationship determined using general linear model.

ß, correlation coefficient; CI, confidence interval; PMH, positive mental health; Gr., grade.

## Discussion

This study investigated the association between sociodemographic characteristics, total PMH and domains. In addition, the study found strong sociodemographic correlates for both the total PMH and domains in patients with mental illnesses who were seen in the outpatient division of a tertiary psychiatric referral hospital in Gauteng province, South Africa.

### Effect of gender

Gender remained a significant (*p* = 0.018) predictor for the total PMH in this study, with women reporting higher PMH compared to men in all domains except for the global effect. Females reporting higher levels of well-being are attributed to the fact that they have stronger social networks and effectively seek out support and assistance. Compared to men, women are more adept at overcoming mental health difficulties and recovering quickly.^14^ In addition, women have higher emotional sensitivity and responsiveness.^15^ A study conducted by Matud^16^ in Spain reported that females scored statistically significant scores as compared to males in the psychological well-being in the personal growth and positive relations with others.

### Effect of education level

This study found that education level was significantly (*p* < 0.001) related to the total PMH and all domains. The linear regression model also identified that the level of education is a predictor of PMH. Respondents with tertiary education reported significantly higher scores across the total PMH and all other domains. Higher education is associated with individuals’ psychological and subjective well-being.^17,18^ Lower education is associated with lower self-reported physical and mental health. These outcomes confirm the association between the level of education and mental health.^19^ Higher education lowers the risk of mental disease and enhances individual’s mental health.^20^ Education leads to careers with higher financial resources and social standing, which enhances people’s quality of life and sense of self, which in turn enhances mental health.^21^

### Effect of employment

Participants who are employed reported significantly (*p* = 0.005) higher scores in the total PMH and all domains except for spirituality. The linear regression analysis also indicated that employment is a predictor of the total PMH and all domains except for the emotional support and spirituality domains. Unemployment can affect individuals’ psychological well-being and other domains of PMH.^22^ Unemployment and not receiving social grants or benefits has an adverse influence on the mental health of the population.^23^ Lack of stable employment is associated with lower mental health. A gradient was established between the lack of stable employment and the deteriorating mental health of the population.^24^ Studies conducted in developed^25,26^ and developing countries^27^ reported that unemployment led to poor mental health because of an increase in the psychosocial burden.

Although married and single participants reported higher scores in most domains, there was no significant (*p* = 0.342) difference in mean scores. Even though more than half of the study population had been on treatment for 4–7 years, there were no significant (*p* = 0.666) differences observed in their PMH scores and domains.

## Conclusions

An investigation of the PMH and its domains and further interventions in treatment approaches can create a positive interest in healthcare workers, which can contribute to better patient outcomes with mental health in South Africa. The findings of this study will hopefully encourage mental health professionals to engage actively to examine the domains of the PMH and to improve individuals’ mental health. Positive mental health research and its domains, as well as additional interventions in treatment approaches can pique the interest of healthcare workers, resulting in better patient outcomes with mental health in South Africa.

### Limitations

This is a cross-sectional study in a comparatively smaller group and was conducted in one tertiary referral psychiatric hospital in Gauteng. Hence, the study results cannot be generalised to the overall population in South Africa or used to establish causal relationships. The study included patients from the outpatient department of the study site. Thus, the study results may not be generalised to in-patients. Despite these limitations, we believe that the results are convincing and, therefore, may provide knowledge for future research in the field of PMH.

### Recommendations

The introduction of South Africa’s *Mental Health Care Act (17 of 2002)* has raised awareness of the public health approach to enhance the mental health of the population. This may require creating awareness among mental healthcare professionals in assessing various domains of PMH and its importance in implementing appropriate treatment strategies. The burden of mental health can be considerably reduced by providing the healthcare workers with the training and resources they need to develop the necessary competences to improve both the mental health of individuals and the population.
